# Quantitative analysis of regional distribution of tau pathology with ^11^C-PBB3-PET in a clinical setting

**DOI:** 10.1371/journal.pone.0266906

**Published:** 2022-04-11

**Authors:** Elham Yousefzadeh-Nowshahr, Gordon Winter, Peter Bohn, Katharina Kneer, Christine A. F. von Arnim, Markus Otto, Christoph Solbach, Sarah Anderl-Straub, Dörte Polivka, Patrick Fissler, Joachim Strobel, Peter Kletting, Matthias W. Riepe, Makoto Higuchi, Gerhard Glatting, Albert Ludolph, Ambros J. Beer, for the Alzheimer’s Disease Neuroimaging Initiative

**Affiliations:** 1 Department of Nuclear Medicine, Medical Radiation Physics, Ulm University, Ulm, Germany; 2 Department of Nuclear Medicine, Medical Center—University of Freiburg, Faculty of Medicine, University of Freiburg, Freiburg, Germany; 3 Department of Nuclear Medicine, Ulm University, Ulm, Germany; 4 Department of Nuclear Medicine, Inselspital Bern—University of Bern, Bern, Switzerland; 5 Department of Neurology, Ulm University, Ulm, Germany; 6 Department of Geriatrics, University Medical Center Göttingen, Göttingen, Germany; 7 Department of Neurology, University Hospital Halle (Saale), Halle, Germany; 8 Psychiatric Services of Thurgovia (Academic Teaching Hospital of Medical University Salzburg), Münsterlingen, Switzerland; 9 Department of Psychiatry and Psychotherapy II, Ulm University, Ulm, Germany; 10 National Institute of Radiological Sciences, Chiba, Japan; 11 German Center for Neurodegerative Diseases (DZNE), Ulm, Germany; Banner Alzheimer’s Institute, UNITED STATES

## Abstract

**Purpose:**

The recent developments of tau-positron emission tomography (tau-PET) enable *in vivo* assessment of neuropathological tau aggregates. Among the tau-specific tracers, the application of ^11^C-pyridinyl-butadienyl-benzothiazole 3 (^11^C-PBB3) in PET shows high sensitivity to Alzheimer disease (AD)-related tau deposition. The current study investigates the regional tau load in patients within the AD continuum, biomarker-negative individuals (BN) and patients with suspected non-AD pathophysiology (SNAP) using ^11^C-PBB3-PET.

**Materials and methods:**

A total of 23 memory clinic outpatients with recent decline of episodic memory were examined using ^11^C-PBB3-PET. Pittsburg compound B (^11^C-PIB) PET was available for 17, ^18^F-flurodeoxyglucose (^18^F-FDG) PET for 16, and cerebrospinal fluid (CSF) protein levels for 11 patients. CSF biomarkers were considered abnormal based on Aβ_42_ (< 600 ng/L) and t-tau (> 450 ng/L). The PET biomarkers were classified as positive or negative using statistical parametric mapping (SPM) analysis and visual assessment. Using the amyloid/tau/neurodegeneration (A/T/N) scheme, patients were grouped as within the AD continuum, SNAP, and BN based on amyloid and neurodegeneration status. The ^11^C-PBB3 load detected by PET was compared among the groups using both atlas-based and voxel-wise analyses.

**Results:**

Seven patients were identified as within the AD continuum, 10 SNAP and 6 BN. In voxel-wise analysis, significantly higher ^11^C-PBB3 binding was observed in the AD continuum group compared to the BN patients in the cingulate gyrus, tempo-parieto-occipital junction and frontal lobe. Compared to the SNAP group, patients within the AD continuum had a considerably increased ^11^C-PBB3 uptake in the posterior cingulate cortex. There was no significant difference between SNAP and BN groups. The atlas-based analysis supported the outcome of the voxel-wise quantification analysis.

**Conclusion:**

Our results suggest that ^11^C-PBB3-PET can effectively analyze regional tau load and has the potential to differentiate patients in the AD continuum group from the BN and SNAP group.

## 1 Introduction

In 2018, the National Institute on Aging and Alzheimer’s Association (NIA-AA) has updated the definition of AD by focusing on biomarkers associated with the pathological processes of Alzheimer’s and excluding the clinical symptoms as diagnostic criteria [1]. The biomarkers that are closely correlated with the hallmarks of AD are amyloid-beta (Aß) and tau. However, the role of neurodegeneration or neuronal injury biomarkers in predicting cognitive decline is also undeniable. The NIA-AA framework therefore suggests the A/T/N biomarker classification scheme in AD and brain aging research, where “A” refers to biomarkers of Aß, “T” stands for biomarkers of tau pathology, and “N” refers to biomarkers of neurodegeneration or neuronal injury [2].

Numerous studies have highlighted the importance of Aß biomarkers [3–6] as well as the combination of Aß and neurodegeneration biomarkers in the pathogenesis of AD [7–9]. More recently due to the introduction of PET ligands for pathologic tau, the investigation of the role of tau pathology has also attracted considerable interest. In terms of regional distributions, Aβ is spread diffusely throughout the neocortex, while tau spreads more selectively across the temporal lobe, association cortices, and finally primary sensorimotor cortices, as summarized in the Braak stage scheme of progressive tau pathology [10, 11]. This progression of tau is closely associated with disease stage and cognitive performance [11].

Several PET-tracers have been developed over the past few years to target tau [12–15]. Among them, the highly affine and specific ^11^C-PBB3 may have the potential to be used in visualizing intracellular tau aggregates [16, 17]. However, little is yet known regarding the diagnostic value of ^11^C-PBB3-PET in a routine setting and on an individual patient level.

Clinical studies using a limited number of patients indicated sensitive detection of tau pathology by ^11^C-PBB3 in patients with AD, with evidence of association between ^11^C-PBB3 uptake and disease progression [18, 19]. The ^11^C-PBB3 distribution among cognitively normal and AD groups could mirror the pathological staging [20]. It was reported that in contrast to a relatively low ^11^C-PIB uptake in the hippocampus as a cortical association area in AD, ^11^C-PBB3 provided a robust signal in this region [18]. A head-to-head comparison of different tau tracers demonstrated that ^11^C-PBB3 is more sensitive to tau aggregations that are correlated with amyloid-beta deposits [21]. Moreover, for ^11^C-PBB3 binding to tau aggregates without evidence for positive amyloid-beta detection has been demonstrated [18, 22].

In this preliminary study, we aim to apply the A/T/N biomarker classification scheme to a population of neurological patients and compare the regional tau deposition by ^11^C-PBB3-PET imaging between patients within the AD continuum, BN individuals and patients with SNAP.

## 2 Materials and methods

### 2.1 Study population

A total of 23 patients (Male: 12; Female: 11; mean age: 66.0 ± 6.6 y; range: 52–75 y) with probable neurodegenerative dementia, who underwent an ^11^C-PBB3-PET imaging session, was pooled from the population database of the Neurology Center in the Ulm University Hospital, Germany. For all patients included in the study, biomarker data on amyloid-beta (^11^C-PIB-PET and/or CSF Aß_42_), tau (^11^C-PBB3-PET) and neurodegeneration (^18^F-FDG-PET and/or CSF t-tau) were available. ^11^C-PIB-PET was available for 17 patients, ^18^F-FDG-PET for 16, magnetic resonance (MR) images for 13, and CSF studies for 11 patients. The study was conducted according to the international Declaration of Helsinki and with the national regulations (German Medicinal Products Act, AMG §13 2b). A written informed consent was obtained from all patients.

To identify potential hypometabolism on ^18^F-FDG-PET images, a set of 102 ^18^F-FDG-PET images from cognitively normal individuals (Male: 69; Female: 81; mean age: 69.7 ± 3.7 y; range: 56–75 y) was selected from the Alzheimer’s Disease Neuroimaging Initiative (ADNI) database (http://adni.loni.usc.edu/). The ADNI was launched in 2003 as a public-private partnership, led by Principal Investigator Michael W. Weiner, MD. The primary goal of ADNI has been to test whether serial magnetic resonance imaging (MRI), positron emission tomography (PET), other biological markers, and clinical and neuropsychological assessment can be combined to measure the progression of mild cognitive impairment (MCI) and early Alzheimer’s disease (AD).

In addition, a set of 17 ^11^C-PIB-PET data and the corresponding MR images (Male: 7; Female: 10; mean age: 73.5 ± 8.7 y; range: 59–85 y), including 9 AD patients and 8 healthy subjects, were also obtained from the ADNI database to create ^11^C-PIB-PET templates.

### 2.2 CSF biomarkers

The CSF samples were collected by lumbar puncture at the Ulm University Hospital, Department of Neurology. In brief, samples were centrifuged and stored at -80°C according to local SOPs and the Aß_42_ and t-tau CSF levels were determined.

### 2.3 Imaging biomarkers

#### 2.3.1 Image acquisition

All PET scans were acquired on a Biograph 40 PET/CT scanner (Siemens Medical Solutions, Erlangen, Germany) and low-dose CT scans were used for attenuation correction. For tau-PET, patients were injected with ^11^C-PBB3 of median 517 MBq (range: 186–925 MBq) and, after a 40 min uptake time period, a PET acquisition was performed for 20 min. For amyloid-PET, patients received a single intravenous bolus injection of median 487 MBq (range: 222–567 MBq) of ^11^C-PIB, followed by a 20 min PET acquisition performed 40 min after injection. For ^18^F-FDG-PET, patients were injected with ^18^F-FDG of 200 MBq (range: 174–221 MBq); after a 30 min uptake time period, a 7 min acquisition was performed. Standard corrections for random coincidences, attenuation, decay and scatter were applied. Emission data were reconstructed in a 200 × 200 × 109 matrix (pixel size = 2.04 mm, slice thickness = 2.03 mm) using the iterative OSEM3D algorithm with both point-spread-function and time-of-flight (PSF+TOF) features, 21 subsets and 4 iterations.

The MR images were acquired with a Prisma 3 T clinical scanner (Siemens Medical Solutions, Erlangen, Germany). T1-weighted images were obtained using a magnetization-prepared rapid acquisition gradient echo (MPRAGE) sequence with the following parameters: repetition time = 2300 ms, echo time = 2.03 ms, inversion time = 900 ms, flip ang1e = 9°, 240 × 256 in plane matrix with a phase field of view of 0.94, 192 slices, and slice thickness of 1.0 mm.

#### 2.3.2 Image processing

All PET images were analyzed with an in-house pipeline in the Matlab software (R2017a, MathWorks, Natick, Massachusetts, USA) that uses the Statistical Parametric Mapping software package (SPM12; www.fil.ion.ac.uk/spm).

Since not all patients had an MRI, there was a necessity for a PET-template-based preprocessing method. Various studies have shown that the spatial normalization using PET templates are highly effective for quantification of hypometabolism and amyloid deposition using PET [23–25]. The feasibility of a PET-based method for the quantification of ^11^C-PBB3 tracer was also evaluated in our previous study [26].

**Fig 1:**
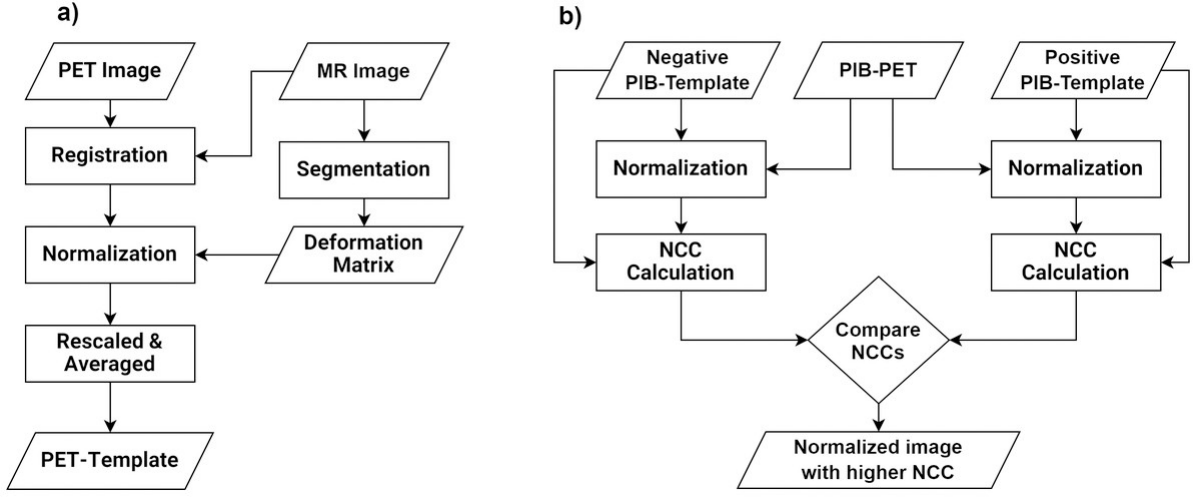
Flowchart of the image processing procedures. a) PET images were co-registered with the corresponding MR images. The SPM unified segmentation algorithm was used to normalize MR images into the *MNI* space. The forward transformation matrices were applied to the PET images. Normalized PET scans were scaled and averaged to generate a PET-template. b) Each ^11^C-PIB-PET patient image was normalized into both positive and negative PIB-templates. The normalized cross-correlation (*NCC*) was calculated between the PIB-templates and normalized ^11^C-PIB-PET images. Normalized ^11^C-PIB-image with higher *NCC* is selected for the rest of the study.

For tau-PET, the ^11^C-PBB3-PET images with available MR scans were co-registered with the corresponding MR images using the normalized mutual information maximization algorithm. The MR images were then aligned with the standard T1-template provided by SPM12 using the unified segmentation-normalization algorithm [27]. The obtained transformation matrices were applied to the corresponding ^11^C-PBB3-PET images to normalize them into the Montreal Neurological Institute (*MNI*) space. Next, the PET images were scaled to the cerebellum and averaged for generation of a ^11^C-PBB3-PET template (Fig 1A). Subsequently, all 23 individual ^11^C-PBB3-PET images were spatially normalized into the ^11^C-PBB3 template using the ‘old normalization’ module of SPM12 [28]. A detailed description of the method can be found in [26].

Since images of amyloid-positive and -negative patients have different activity distribution patterns, adaptive template methods have been suggested for PET-based amyloid quantification [23]. Nine positive and eight negative ^11^C-PIB-PET images with available MRI from the ADNI database were normalized into the *MNI* space according to the procedure described above (Fig 1A). Positive and negative images were then averaged to generate positive and negative templates, respectively. Every ^11^C-PIB-PET patient image was non-rigidly normalized into both positive and negative templates using the ‘old normalization’ module of SPM12. The normalized cross-correlation (*NCC*) was calculated between the ^11^C-PIB-templates and all spatially normalized ^11^C-PIB-PET images as follows [23]:

$${NCC}_{z}=\frac{1}{n}\frac{\sum_{x,y}\left(I(x,y)-\bar{I})(T(x,y)-\bar{T}\right)}{\sigma_{I}\sigma_{T}}$$

where *NCC*_*z*_ means the *NCC* on each axial slice (z), *n* stands for the number of pixels per slice, *σ* is the standard deviation, *T* and *I* represent the template and ^11^C-PIB-PET images, respectively. The template with higher *NCC* was adopted (Fig 1B).

The ^18^F-FDG-PET images from the ADNI dataset included 6 frames of 5 min duration from 30 to 60 min post injection. The first frame of these images was comparable to the ^18^F-FDG-PET images used in this study, with an acquisition time of 7 min. Therefore, only first frame of the ADNI ^18^F-FDG-PET images was used for the voxel-wise SPM analysis. In addition, the ADNI images were filtered with a scanner-specific filter function to produce images of a common resolution of 8 mm FWHM, the approximate resolution of the lowest resolution scanners used in ADNI. The effective spatial resolution in our brain ^18^F-FDG-PET scans after iterative reconstruction using a 5 mm Gaussian filter was also approximately 8 mm FWHM. For normalization of the ^18^F-FDG-PET images, the dementia-specific FDG-PET template developed by Della Rosa *et al*. was used [29, 30]. This template was built by averaging the ^18^F-FDG-PET images of 50 healthy controls and 50 patients with dementia (http://inlab.ibfm.cnr.it/inlab/PET_template.php). All normalized ^18^F-FDG-PET scans were then smoothed with an isotropic Gaussian kernel of 8 mm FWHM for single-subject voxel-wise analysis, as suggested in [29, 31, 32].

### 2.4 Biomarkers

#### 2.4.1 CSF biomarkers

The CSF biomarker profile was considered abnormal if the CSF Aβ_42_ level was below 600 ng/L (A^+^) and the CSF t-tau value was higher than 450 ng/L (N^+^) [33].

#### 2.4.2 Imaging biomarkers

All ^11^C-PIB-PET and ^18^F-FDG-PET images were evaluated with visual assessment by two experienced nuclear medicine physicians (P.B. and A.J.B.).

The Hammers grey-matter-masked probabilistic brain map was used to calculate regional PET values of the grey matter for each patient [34, 35]. Median PET values in each volume of interest (VOI) were then divided by median uptake in cerebellar crus grey matter to create standardized uptake value ratios (SUVRs). To classify the ^11^C-PIB-PET scans as positive/negative (A^+^ /A^-^), the global PIB retention ratios were calculated from the volume-weighted average SUVRs of bilateral frontal, precuneus/posterior cingulate gyri, anterior cingulate gyri, superior parietal and lateral temporal VOIs. Using visually established amyloid positivity as the gold standard, a receiver operating characteristics (ROC) analysis was performed on the global SUVR values to determine the optimal threshold for classification of A^+^ and A^-^. The cutoff point was computed from the ROC curve at the point with the largest Youden’s index [36]. A leave-one-out-cross-validation (LOOCV) was applied to evaluate the accuracy of the cutoff point.

^18^F-FDG-PET biomarker positivity (N^+^) was defined using visual inspection combined with the optimized single-subject SPM analysis, as recommended by the common practice guideline for brain ^18^F-FDG-PET in patients with dementing disorders [37]. The preprocessing steps for the normalized ^18^F-FDG-PET images for optimal single-subject statistical analysis have been described elsewhere [29, 31, 32]. Each ^18^F-FDG-PET patient image was evaluated with respect to the 102 healthy controls via the two sample t-test in SPM. All analyses were controlled for age and sex. Clusters of hypometabolism were considered significant when they were present in the typical VOIs, which are more susceptible to the neurodegenerative dementia, with a minimum extent of 100 voxels and surviving at *p* < 0.05 FWE corrected threshold at a voxel level. The hypometabolism pattern, obtained with single-subject SPM analysis, supports the visual inspection to classify the ^18^F-FDG-PET images.

### 2.5 Group classification

Categorization into diagnostic groups was made based on the imaging or CSF biomarkers by applying the NIA-AA criteria [1]. The patients were classified into three groups using Amyloid (A) and neurodegeneration or neuronal injury biomarkers (N). Six patients were identified as BN (A^-^T*N^-^), ten SNAP (A^-^T*N^+^) and seven within the AD continuum (A^+^T*N^-^ [n = 2] or A^+^T*N^+^ [n = 5]). The absent biomarker group in the classification process is labeled with an asterisk (*).

Among the seven patients within the AD continuum, three had a diagnosis of typical AD, three logopenic primary progressive aphasia (PPA) and one undetermined. Among the patients categorized as SNAP, four had non-fluent PPA, two semantic PPA, three corticobasal dementia (CBD) and one behavioral frontotemporal dementia (bv-FTD). Among the individuals identified as BN, one had progressive supranuclear palsy (PSP), one non-fluent PPA, one vascular Parkinson (VP) and three undetermined.

### 2.6 Statistical analysis

#### 2.6.1 Voxel-wise analyses

Before group comparisons, a grey matter probability map from the Hammers probabilistic brain atlas was used to mask the ^11^C-PBB3-PET images for grey matter. Then subjects within the AD continuum were compared with BN and SNAP groups using a voxel-wise two-tailed student’s t-test, assuming independence and unequal variances. An explicit mask was used to restrict the analyses only to within-brain voxels. All ^11^C-PBB3-PET images were intensity-normalized to the cerebellum as reference region. Due to a relatively small sample size of this study and to increase the sensitivity of the analysis, the threshold of *p* < 0.01 under uncorrected statistics at voxel level was applied. However, only clusters surviving at *p* < 0.05 (FWE corrected) and for cluster extent of k > 100 are reported.

#### 2.6.2 Atlas-based analyses

To evaluate whether the signal extracted from the predefined VOIs was different between patients within the AD continuum and two other groups, an atlas-based analysis was performed. The Hammers probabilistic brain atlas which contains 95 regions was combined into the following meta-VOIs, which are known to be associated with tau deposition in AD: the medial temporal lobe including the hippocampus, parahippocampal gyrus and amygdala; the temporal lobe including the inferior, middle, anterior, posterior and superior temporal gyri and fusiform; the frontal lobe including the inferior, middle, and superior frontal gyri, orbitofrontal gyrus, rectus and precentral gyrus; the occipital lobe including the lateral remainder of occipital cortex, lingual gyrus and cuneus; the parietal lobe including the superior parietal, postcentral, supramarginal and angular gyri; anterior cingulate cortex; posterior cingulate cortex and global cortical calculated by the volume-weighted average SUVRs of the above meta-VOIs.

Statistical analyses were performed using the R Statistical Software version 3.6.3 (the R Project for statistical computing, available at https://www.r-project.org/). Due to the limited number of patients, non-parametric tests were used for analysis. The SUVR values in the meta-VOIs were compared between groups using the non-parametric one-way analysis of variance (ANOVA) followed by Bonferroni post hoc test. A *p*-value < 0.05 was considered statistically significant. The effect sizes for the discrimination between groups were calculated using Cliff’s Delta (delta), a non-parametric effect size measure which ranges between -1 and +1 [38]. An effect size of -1 or +1 shows a perfect separation between two groups, whereas an effect size of 0 indicates a complete overlap between groups. The magnitude of the effect sizes is assessed using the thresholds provided in [39], where |delta| < 0.33 indicates small effect sizes, 0.33 < |delta| < 0.47 represents medium effect sizes and |delta| > 0.47 large effect sizes.

## 3 Results

### 3.1 Categorization of scans

The global SUVR cutoff value for amyloid positivity, that provided the highest Youden’s index with a sensitivity and specificity of 100%, was 1.58. The leave‐one‐out cross validation resulted in a minor reduction of average classification accuracy to 94% (AUC: 0.99, sensitivity: 100%, specificity: 0.86%). In the semi-quantitative scan classification, 41% (7/17) of the ^11^C-PIB-PET images were determined as amyloid-positive (A^+^). By visual inspection, 75% (12/16) of the ^18^F-FDG-PET images were defined as neurodegeneration-positive (N^+^).

Demographics of the AD continuum, SNAP and BN patients are presented in Table 1. There were no significant differences in age and sex between groups. The cognitive performance tended to be lower in the AD continuum and SNAP groups compared to the BN group. CSF levels of Aβ_42_ and t-tau were also recorded, when available. There was no significant difference in the recorded CSF levels between the groups. However, the statistical power may be limited due to the small sample size.

**Table 1:** Cohort demographics.

	AD continuum	SNAP	BN
	(A^+^T*N^-^/A^+^T*N^+^)	(A^-^T*N^+^)	(A^-^T*N^-^)
n	7	10	6
Age (y)	66 ± 5	66 ± 6	63 ± 9
Sex (F/M)	3/4	5/5	3/3
MMSE (median, range)	24 (14–27)	25 (17–29)	27 (21–30)
No. with FDG-PET/CT	4	9	3
Global PIB-PET/CT	2.21 ± 0.25 (n = 7)	1.30 ± 0.17 (n = 6)	1.36 ± 0.06 (n = 4)
CSF Aß_42_ (ng/L)	499 ± 169 (n = 3)	1065 ± 588 (n = 4)	841 ± 218 (n = 4)
CSF t-tau (ng/L)	440 ± 230 (n = 3)	462 ± 213 (n = 4)	267 ± 51 (n = 4)

AD: Alzheimer’s Disease; SNAP: suspected non-AD pathophysiology; BN: biomarker-negative; A: amyloid Aß_42_ biomarkers; T: tau pathology biomarkers; N: neurodegeneration or neuronal injury biomarkers; n: number of patients; y: years; M: male; F: female; MMSE: Mini-Mental State Examination (0–30, 30 = perfect score).

### 3.2 Voxel-wise analyses

**Fig 2:**
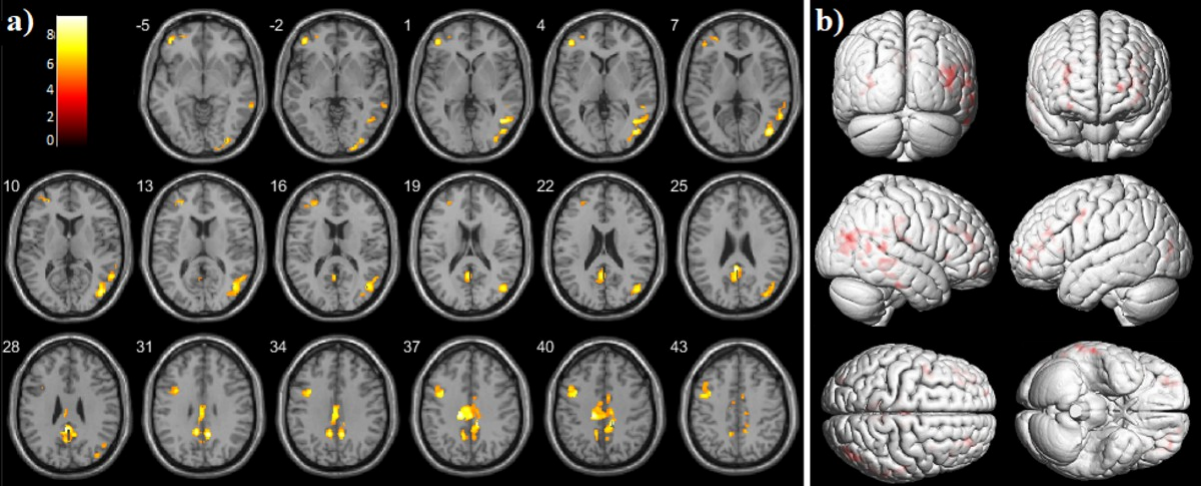
The voxel-wise SPM analysis of increased tau uptake. a) ^11^C-PBB3-SUVRs in the AD continuum patients in contrast to the BN group. The threshold of *p* < 0.05 under FWE corrected statistics at cluster level was applied. The color bar values indicate the value of the T-statistics. b) Surface rendering shows the volume where ^11^C-PBB3-SUVRs were increased in the AD continuum in comparison to BN patients.

The SPM analysis showed that patients within the AD continuum had significantly higher ^11^C-PBB3 uptake than BN patients in the cingulate gyrus and temporo-parieto-occipital junction as well as in the frontal region (Fig 2 and Table 2).

**Table 2:** Voxel-wise comparing of the ^11^C-PBB3 uptake between patients within the AD continuum (n = 7) and BN individuals (n = 6). Cerebellar crus grey matter was used as a reference region to calculate the SUVRs.

Region	Cluster size (voxels)	SUVR (^11^C-PBB3)		
		AD continuum	BN	Effect size
		Median (IQR)	Median (IQR)	(delta)
Cingulate gyrus (anterior & posterior)	758	1.26 (0.07)	0.93 (0.03)	1.00
Temporo-parieto-occipital junction	658	1.24 (0.09)	0.92 (0.05)	1.00
Superior Frontal	257	1.16 (0.05)	0.86 (0.05)	1.00

VOI: volume-of-interest; SUVR: standardized-uptake-value-ratio.

**Fig 3:**
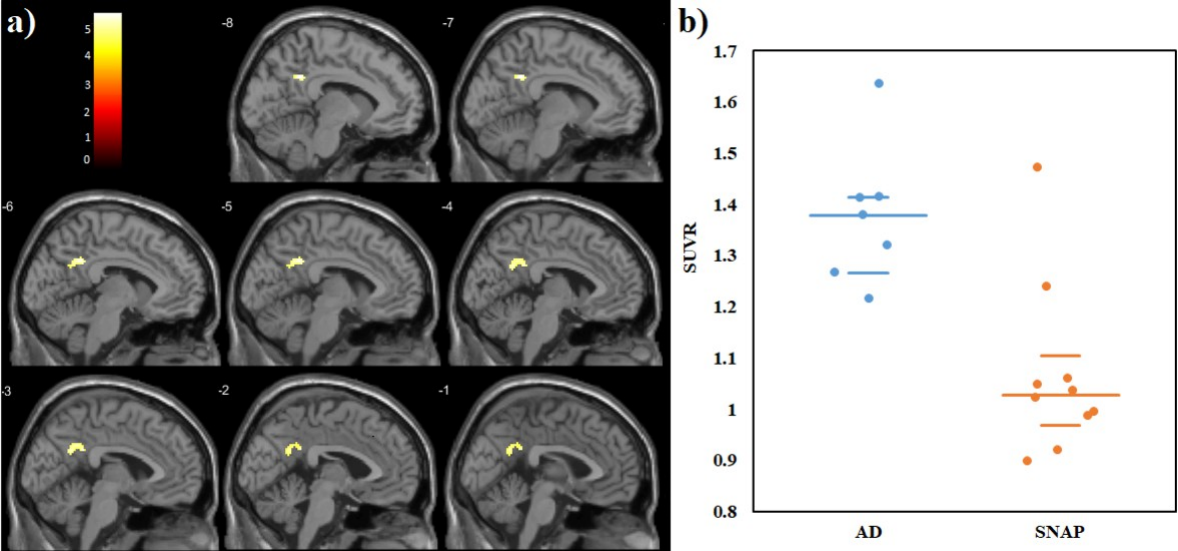
Regional statistical parametric mapping analysis of tau depositions. a) ^11^C-PBB3-SUVRs in the AD continuum patients in contrast to the SNAP group. The threshold of *p* < 0.05 under FWE corrected statistics at cluster level was applied. The color bar values indicate the value of the T-statistics. b) ^11^C-PBB3-SUVRs values from the posterior cingulate shows a contrast between patients within the AD continuum and the SNAP group. The horizontal lines are the median and the 25^th^ and 75^th^ percentiles.

Comparing the SUVRs between AD continuum and SNAP patients, AD continuum patients had a slightly increased ^11^C-PBB3 uptake over the posterior cingulate cortex (median and interquartile range [IQR] of 1.38 [0.12] vs. 1.03 [0.17]; 289 voxels; delta = 0.80). The variability and overlap in the SUVR values from the posterior cingulate cortex between both patient groups are presented in Fig 3.

### 3.3 Atlas-based analyses

The atlas-based quantitative analysis of ^11^C-PBB3-PET images revealed that the SUVR values of the temporal, frontal, parietal, occipital lobes and posterior cingulate cortex were significantly higher in the AD continuum group than in the BN group (Table 3; *p* < 0.01 and delta ≥ 0.95 for all). By regional analysis a significant increase of ^11^C-PBB3-SUVRs in patients within the AD continuum as compared to the SNAP group in the posterior cingulate (Table 3; *p* = 0.04; delta = 0.72) was demonstrated. The SUVRs of the predefined meta-VOIs between SNAP and BN patient groups indicated no significant differences.

**Table 3:** The median ^11^C-PBB3-SUVR values of meta-VOIs with interquartile ranges (IQR) and Cliff’s Delta effect sizes (delta) for the three cohorts. Patients within the AD continuum were compared with SNAP and BN patient groups using the non-parametric one-way ANOVA followed by Bonferroni post hoc test.

Region	SUVR (^11^C-PBB3)				
	AD continuum	BN	Effect size	SNAP	Effect size
	Median (IQR)	Median (IQR)	(delta)	Median (IQR)	(delta)
Medial Temporal	1.00 (0.06)	0.96 (0.06)	0.48	0.93 (0.10)	0.42
Temporal	1.11 (0.03)	0.95 (0.04)*	1.00	0.94 (0.15)	0.60
Frontal	1.07 (0.05)	0.87 (0.06)*	1.00	0.88 (0.18)	0.66
Parietal	1.05 (0.08)	0.92 (0.01)*	0.95	0.91 (0.16)	0.57
Occipital	1.11 (0.07)	0.98 (0.04)*	0.95	0.99 (0.13)	0.66
Anterior Cingulate	1.09 (0.07)	0.89 (0.06)†	0.86	0.90 (0.17)	0.60
Posterior Cingulate	1.18 (0.14)	0.98 (0.08)*	1.00	0.97 (0.14)†	0.72
Global	1.09 (0.05)	0.92 (0.03)*	1.00	0.92 (0.16)	0.62

† if *p* < 0.05 and

* if *p* < 0.01.

**Fig 4:**
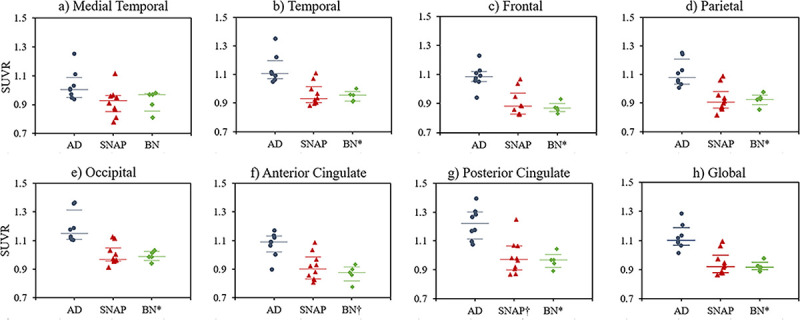
Scatter plots showing ^11^C-PBB3-SUVRs in the three patient groups. The variety of tau-SUVRs in the predefined meta-VOIs of a) the medial temporal lobe b) temporal lobe, c) frontal cortex, d) parietal cortex, e) occipital cortex, f) anterior cingulate, g) posterior cingulate and h) global cortical are presented for the BN, SNAP and patients within the AD continuum. The latter group was compared with the SNAP and BN groups using the non-parametric ANOVA following by Bonferroni post hoc test. The corresponding *p-*values are indicated with symbols († if *p* < 0.05 and * if *p* < 0.01). The horizontal lines are the median and the 25^th^ and 75^th^ percentiles.

The variability and overlap in the ^11^C-PBB3-SUVR values from the predefined meta-VOIs for all three patient groups are presented in Fig 4. There was less overlap in the ^11^C-PBB3 uptake between patients in the AD continuum and BN groups for all meta-VOIs except for the medial temporal region (Fig 4A; *p* = 0.5; delta = 0.48). In contrast, tau pathology in the SNAP group was similar to that of the BN group (*p* > 0.2 for all VOIs).

## 4 Discussion

To date, the results of only few clinical trials with ^11^C-PBB3 tau tracer are available [18, 19, 22, 40]. Therefore, this is an area where more research is needed to validate the diagnostic value of ^11^C-PBB3-PET. In this study, the tau deposition using ^11^C-PBB3-PET in the AD continuum, SNAP and BN patients was assessed. The quantitative analyses showed a higher global SUVR and SUVR in several cortical regions in patients within the AD continuum than in BN patients. Furthermore, the SUVR in the posterior cingulate was significantly higher in the AD continuum patients than in SNAP patients. The results indicate that ^11^C-PBB3-PET is indeed a noninvasive biomarker for tau deposition.

The main strength of this study is to provide semi-automated techniques to analyse the PET data. The PET-based quantitative method was used to quantify tau-PET scans [26]. For amyloid-PET quantification, the adaptive template method was utilized due to the different activity distribution patterns in amyloid-positive and -negative patients [23]. The optimized single-subject SPM approach was used to support the visual inspection of ^18^F-FDG-PET images [30]. Visual assessment of PET scans is commonly used in many nuclear medicine facilities. However, the automated and semi-automated quantitative methods can significantly improve the detection and comparative assessment. Furthermore, the non-specific binding of radiotracers makes the detection of cerebral cortical binding challenging for the human eye. This process could be even more difficult for ^11^C-PBB3-PET images due to the lower specific binding of the ^11^C-PBB3 compared to other tau tracers [41]. Nevertheless, since the automated analysis of ^18^F-FDG-PET is still a matter of debate [42], the visual assessment of ^18^F-FDG-PET images was considered as the preferred method in this study.

Comparing the regional ^11^C-PBB3-SUVR values between patients within the AD continuum and BN, higher SUVRs were noted over the cingulate gyrus, temporo-parieto-occipital junction and frontal regions, which were similar to previous studies. Maruyama *et al*. reported that in the patients with AD, ^11^C-PBB3 accumulation was most frequently observed in the limbic system and gradually spread into the temporal, parietal and frontal regions that correspond to Braak stages V-VI [18]. However, this study has included only a small number of patients (3 AD vs. 3 cognitively normal individuals). Kimura *et al*. evaluated the feasibility of kinetic model-based approaches to quantify tau binding using ^11^C-PBB3-PET and blood data [40]. They found that the reference tissue and the dual-input model binding parameters discriminate effectively normal controls from patients with AD. Terada investigated the uptake of ^11^C-PBB3 in participants with early AD [43]. He reported notable differences in tracer uptake in the temporo-parietal junction of AD patients compared to healthy controls. In our study, all BN participants were classified as Braak stage I/II, which will be explained below in more detail. Patients within AD continuum showed elevated tracer retention in regions corresponding to Braak stage III/IV. Although the patients in this study are mostly in the mild to moderate dementia category, the gradual spread of ^11^C-PBB3 accumulation is clearly observed in the parietal and frontal lobes (Braak stage V/VI). Our results therefore add further evidence supporting the hypothesis that the ^11^C-PBB3 tau ligand is able to discriminate cognitively normal patients from those within the AD continuum.

Patients within the AD continuum had a higher cortical ^11^C-PBB3-SUVR than SNAP patients in various brain regions (Table 3). However, due to the small sample size and large standard deviation of the regional SUVR values in the SNAP group, no significant differences were found between two groups, except for the posterior cingulate area (Figs 3 and 4). The wide IQR of regional SUVRs in the SNAP patients can be explained by the heterogeneity of the dementia subtypes in this group. Moreover, different dementia subtypes have been associated with different pathological hallmarks, often showing AD co-pathology. Several studies have reported that the non-AD patients with AD co-pathology are more likely to be classified as AD [44–46]. This may explain the overlap of SNAP and AD continuum group in the current work.

The SNAP group was generally intermediate with regard to the distribution of ^11^C-PBB3 uptake relative to the BN and AD continuum group. On an individual basis, two of ten SNAP patients (CBD [n = 1] and semantic PPA [n = 1]) showed high ^11^C-PBB3 uptake in cortical regions that was compatible to AD patient uptakes. The other SNAP subjects had ^11^C-PBB3-SUVR values in the same range as BN patients. Both voxel-wise and atlas-based analyses revealed no significant difference between the SNAP and the BN patient groups (Fig 4).

Although, there was an increasing tendency of ^11^C-PBB3 uptake in the AD continuum group compared to the BN group in the medial temporal lobe, no significant differences for the ^11^C-PBB3-SUVR values were found among the three patient groups (Fig 4A). This finding is compatible with previous studies demonstrating that neurofibrillary tangles around the medial temporal cortex are indistinguishable from those of AD in normal cognitive or SNAP elderly patients [11, 19]. Recently, the new term "primary age-related tauopathy" (PART) has been proposed for such a pathological condition [47].

Both, atlas-based analysis and voxel-wise analysis were performed in this study. Taking the small sample sizes into account, the use of two methods led to more reliable results. The atlas-based approach was also investigated by a previous ^11^C-PBB3-PET study [19]. In this method, the VOI’s signal is typically computed by averaging over all voxel signals in a given VOI. However, the sub-region of the brain, showing statistically significant signals, does not necessarily include the whole voxels within the predefined VOIs. This average over all voxels can thus affect the effect sizes. Conversely, the voxel-wise analysis enables to detect significant signals anywhere between distinct VOIs in the whole brain. As shown in Tables 2 and 3, observed effect sizes based on the atlas-based approach are smaller than those of the voxel-wise method. Despite this observation, the voxel-wise quantitative analysis of ^11^C-PBB3-PET images supported the outcome of the atlas-based analysis.

Although A/T/N biomarker classification scheme originally emerged as a research framework, applying A/T/N to our cohort of patients revealed a good but partial correspondence to the clinical diagnosis. Clinically AD-diagnosed patients (n = 3) and logopenic PPA (n = 3), which is typically associated with AD pathology, were in the AD continuum. Among SNAP patients, 3 out of 16 were identified as BN.

The main limitation of the current study is the lack of cognitively normal individuals. In addition, four spatiotemporal subtypes of tau pathology spread in AD has been recently proposed: limbic-predominant phenotype, parietal-dominant and medial temporal lobe (MTL)-sparing phenotype, predominant posterior occipitotemporal phenotype and asymmetric temporoparietal phenotype [48]. Both, the heterogeneity in AD and lack of cognitively normal individuals could underestimate between-group differences (AD vs. BN and AD vs. SNAP), leading to false-negative results. However, they would not hamper the positive results presented in this study. In group classification, the use of CSF data in the absence of PET images may also be a limitation. Discordance between imaging and CSF biomarkers can cause different positive/negative labels for the same patient. In some situations, discordance in positive/negative labels between an imaging and CSF biomarker is simply due to the borderline cases or non-optimal cutoff values. Excluding patients with a CSF value within ±10% of the cutoff value could reduce this limitation. In this study, there were no patients with CSF values within ±10% of the cut-off values. This supports the validity of combining PET and CSF data for amyloid and neurodegenerative biomarker groups. Moreover, the cutoff-calculation approach for amyloid positivity was data dependent and a larger sample size covering a wide spectrum of cases is needed to yield a more accurate result. However, the LOOCV indicated the stability of the calculated cutoff value in this dataset.
